# Pembrolizumab for treating advanced urothelial carcinoma in patients with impaired performance status: Analysis of a Japanese nationwide cohort

**DOI:** 10.1002/cam4.3863

**Published:** 2021-05-01

**Authors:** Katsuhiro Ito, Takashi Kobayashi, Takahiro Kojima, Kensuke Hikami, Takeshi Yamada, Kosuke Ogawa, Kenji Nakamura, Naoto Sassa, Akira Yokomizo, Takashige Abe, Kazunari Tsuchihashi, Shuichi Tatarano, Junichi Inokuchi, Ryotaro Tomida, Maki Fujiwara, Atsushi Takahashi, Kazumasa Matsumoto, Kosuke Shimizu, Hiromasa Araki, Ryoma Kurahashi, Yu Osaki, Yu Tashiro, Masayuki Uegaki, Osamu Ogawa, Hiroshi Kitamura, Hiroyuki Nishiyama

**Affiliations:** ^1^ Department of Urology Kyoto University Hospital Kyoto Japan; ^2^ Department of Urology Ijinkai Takeda General Hospital Kyoto Japan; ^3^ Department of Urology National Hospital Organization Kyoto Medical Center Kyoto Japan; ^4^ Department of Urology Kyoto University Graduate School of Medicine Kyoto Japan; ^5^ Department of Urology University of Tsukuba Ibaraki Japan; ^6^ Department of Urology Japanese Red Cross Wakayama Medical Center Wakayama Japan; ^7^ Department of Urology Kyoto Prefectural University of Medicine Kyoto Japan; ^8^ Department of Urology Kobe City Nishi‐Kobe Medical Center Hyogo Japan; ^9^ Department of Urology Japanese Red Cross Osaka Hospital Osaka Japan; ^10^ Department of Urology Nagoya University Aichi Japan; ^11^ Department of Urology Harasanshin Hospital Fukuoka Japan; ^12^ Department of Urology Hokkaido University Graduate School of Medicine Sapporo Japan; ^13^ Department of Urology Japanese Red Cross Otsu Hospital Shiga Japan; ^14^ Department of Urology Kagoshima University Kagoshima Japan; ^15^ Department of Urology Graduate School of Medical Sciences Kyushu University Fukuoka Japan; ^16^ Department of Urology Shikoku Cancer Center Ehime Japan; ^17^ Department of Urology Tenri Hospital Nara Japan; ^18^ Department of Urology Hakodate Goryoukaku Hospital Hokkaido Japan; ^19^ Department of Urology Kitasato University Kanagawa Japan; ^20^ Department of Urology Kyoto Katsura Hospital Kyoto Japan; ^21^ Department of Urology Kumamoto University Kumamoto Japan; ^22^ Department of Urology National Hospital Organization Himeji Medical Center Hyogo Japan; ^23^ Department of Urology Tazuke Kofukai Medical Research Institute Kitano Hospital Osaka Japan; ^24^ Department of Urology Toyooka Hospital Hyogo Japan; ^25^ Department of Urology Toyama University Toyama Japan

**Keywords:** bladder cancer, immunotherapy, pembrolizumab, performance status, transitional cell carcinoma, urothelial carcinoma

## Abstract

**Background:**

The benefits of pembrolizumab in patients with advanced urothelial carcinoma (UC) and impaired performance status (PS) remain unknown. This study assessed the safety and efficacy of pembrolizumab in patients with platinum‐refractory UC and Eastern Cooperative Oncology Group PS ≥2 to identify which subgroups may benefit from this drug.

**Methods:**

This retrospective nationwide cohort study collected clinicopathological information for 755 patients from 59 institutions. The overall response rate (ORR) and overall survival (OS) were compared among the patients with PS 0–1, 2, and 3–4. Multivariate analysis was conducted to identify factors predicting OS in patients with PS ≥2.

**Results:**

The numbers of patients with PS 0–1, 2, and 3–4 were 602, 98, and 55, respectively; the ORRs in these groups were 29.5, 15.3, and 9.1%, respectively, and the median OS times were 14.3, 3.1, and 2.4 months, respectively. In multivariate Cox regression analysis, a neutrophil–lymphocyte ratio (NLR) ≥3.5 (hazard ratio [HR] = 1.897) and liver metastasis (HR = 2.072) were associated with OS in the PS ≥2 subgroup. The median OS of patients with PS ≥2 without either risk factor was 6.8 months, compared with 3.1 months for patients with one risk factor and 2.3 months for patients with both risk factors.

**Conclusions:**

PS ≥2 portended worse ORR and OS than PS ≤1 despite a comparable safety profile. Among the patients with impaired PS, patients with NLR <3.5 and no liver metastasis may most greatly benefit from pembrolizumab therapy.

## Graphical abstract

Among patients who received pembrolizumab to treat urothelial carcinoma, those with Eastern Cooperative Oncology Group performance status ≥2 exhibited worse overall response rates and overall survival than those with performance status ≤1 despite their comparable safety profiles. Among the patients with impaired performance status, those with a neutrophil–lymphocyte ratio <3.5 and no liver metastases may most greatly benefit from pembrolizumab.

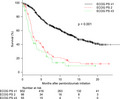

## 1 INTRODUCTION

The advent of immune checkpoint inhibitors (ICIs) has revolutionized the treatment of urothelial carcinoma (UC). Pembrolizumab, an anti‐programmed death 1 antibody, was the first drug to significantly improve overall survival (OS) after platinum‐based chemotherapy, as reported in the KEYNOTE‐045 phase 3 randomized controlled trial.^1^ The objective response rate (ORR) was 21%, including a complete response (CR) rate of 7%, providing hope to patients with advanced UC. Because the phase 3 trial had strict inclusion criteria, additional data are needed for patients outside the trial to extrapolate this promising result to the real world.

The Eastern Cooperative Oncology Group (ECOG) performance status (PS) is a strong prognostic factor for survival in patients with UC.^2^, ^3^ Patients with PS ≥2 are considered unfit for systemic chemotherapy because of the limited efficacy and high toxicity.^4^ ICIs have been reported to have low risk of systemic toxicities such as anorexia or myelosuppression than chemotherapy,^1^ and physicians may have a low threshold to administer ICIs to patients with impaired PS. Meanwhile, clinicians face a difficult decision concerning whether to administer treatment to patients with little possibility of positive outcomes. There is sparse evidence to support the use of ICIs in patients with impaired PS. KEYNOTE‐045 and other phase 3 trials of ICI therapy included extremely small numbers of patients with PS 2. No studies have focused on patients with PS 3 or 4, who are not enrolled in clinical trials. Despite the lack of evidence, the use of pembrolizumab in such patients who are at the end of life, so‐called “desperation oncology,” is increasing.^5^

Our group reported nationwide real‐world data for patients who received pembrolizumab for UC (Japan Urological Oncology Group database) and identified several prognostic factors including PS.^6^ The study illustrated that approximately 20% of patients with metastatic UC who received pembrolizumab had poor PS (≥2) in real‐world practice. This study evaluated the efficacy and safety of pembrolizumab in patients with poor PS. Additionally, we aimed to identify patients who would benefit from pembrolizumab treatment despite impaired PS.

## 2 MATERIALS AND METHODS

### 2.1 Patients and data collection

This multi‐institutional study was approved by the institutional review board (anonymized during review process, approved in June 2018) followed by the review boards of all 59 participating institutions. This study conformed to the Declaration of Helsinki. We retrospectively analyzed the outcomes of 758 patients with UC who received pembrolizumab from August 2015 to December 2019 in a Japanese nationwide cohort. This cohort included three patients who received pembrolizumab in clinical trials before approval by Japan's Pharmaceuticals and Medical Devices Agency, while all the remaining patients received pembrolizumab after the approval. Data were collected through the end of 2019 for all patients who could be followed. Patients were excluded if pembrolizumab was administered in the neoadjuvant/adjuvant setting (n = 2) or if platinum chemotherapy was not previously received (n = 1).

The person in charge of each facility reported ECOG PS at the initial diagnosis as well as pembrolizumab administration based on the patient records. The response was evaluated by each person in charge using Response Evaluation Criteria in Solid Tumors version 1.1. ORR was calculated as the proportion of patients with CR or partial response. Treatment‐related toxicity was assessed using Common Terminology Criteria for Adverse Events (CTCAE) version 4.03.

The parameters evaluated in this study included age, sex, smoking status, primary site of UC, histological subtype, prior surgery, number of prior chemotherapy regimens, interval since prior chemotherapy, laboratory variables (hemoglobin, albumin, neutrophil–lymphocyte ratio [NLR]) at pembrolizumab initiation, and site of metastasis at pembrolizumab initiation.

### 2.2 Analysis method

All statistical analyses were performed using EZR (Saitama Medical Center, Jichi Medical University), which is a graphical user interface for R (The R Foundation for Statistical Computing, version 2.13.0).^7^ It is also a modified version of R commander (version 1.6–3), which includes statistical functions for biostatistics.

The Cochran–Armitage test and Jonckheere–Terpstra test were used to examine trends in categorical and continuous data, respectively. Logistic regression analysis was used for univariate and multivariate analyses to identify prognostic factors for ORR. Progression‐free survival (PFS) and OS were assessed using the Kaplan–Meier method with the log‐rank trend test. Univariate and multivariate Cox proportional hazard models were used to predict prognosis. The prognostic factors in the entire cohort (hemoglobin, NLR, interval since prior chemotherapy, presence of liver metastasis), as reported elsewhere,^6^ and baseline data were included in the analysis. The variables deemed significant at *p* < 0.05 were included in the multivariate analysis. The optimal cutoff for continuous variables (hemoglobin, albumin, NLR, and number of metastatic organs) was determined using receiver operating characteristic curve analysis. All tests were two‐sided, and *p* < 0.05 indicated statistical significance.

## 3 RESULTS

### 3.1 Patients

The characteristics of the patients stratified by PS (0–1, 2, and 3–4) are presented in Table 1. The numbers of patients in the PS 0–1, 2, and 3–4 subgroups were 602 (79.7%), 98 (13.0%), and 55 (7.3%), respectively. Poor PS was significantly associated with a short interval since prior chemotherapy (<90 days, *p* for trend =0.028), lower hemoglobin levels (*p* for trend <0.001) lower albumin levels (*p* for trend <0.001), higher NLRs (*p* for trend <0.001), the presence of bone, liver, peritoneal, and brain metastasis (*p* for trend <0.001, <.0001, 0.002, and <0.001, respectively), and the number of metastatic organs (*p* for trend <0.001). Age, sex, smoking status, the primary site of UC, prior local treatment, and the number of prior chemotherapy regimens did not significantly differ among the subgroups.

**TABLE 1:** Patient characteristics stratified by performance status

	ECOG PS =0–1 n = 602	ECOG PS =2 n = 98	ECOG PS =3–4 n = 55	*p* for trend
Age, year	72.09 [66.30, 77.23]	72.01 [66.49, 76.21]	70.18 [63.50, 75.72]	0.221
Sex, male	456 (75.7)	72 (73.5)	40 (72.7)	0.520
Current or past smoker	340 (59.1)	61 (63.5)	29 (58.0)	0.783
Primary site, bladder	300 (49.8)	52 (53.1)	30 (54.5)	0.402
Variant histology	52 (8.6)	12 (12.2)	6 (10.9)	0.314
Prior cystectomy or nephroureterectomy	347 (57.6)	47 (48.0)	30 (54.5)	0.221
Number of prior chemotherapy				0.152
Adjuvant/NAC	95 (15.8)	13 (13.3)	5 (9.1)	
1 (± adjuvant/NAC)	362 (63.5)	58 (59.2)	38 (69.1)	
2 (± adjuvant/NAC)	108 (17.9)	19 (19.4)	7 (12.7)	
≥3 (± adjuvant/NAC)	37 (6.1)	8 (8.2)	5 (9.1)	
<90 days after prior chemotherapy	273 (45.3)	52 (53.1)	32 (58.2)	0.028*
Hemoglobin, g/dl	10.9 [9.5, 12.1]	10.10 [9.03, 11.07]	9.00 [8.30, 9.85]	<0.001*
Albumin, g/dl	3.80 [1.30, 5.20]	3.30 [1.03, 4.60]	2.80 [1.40, 4.30]	<0.001*
NLR	3.1 [2.1, 4.6]	4.30 [3.14, 7.37]	6.55 [3.87, 12.57]	<0.001*
Lymph nodes metastasis	409 (67.9)	70 (71.4)	32 (58.2)	0.369
Visceral metastasis				
Lung	236 (39.2)	43 (43.9)	22 (40)	0.606
Bone	99 (16.4)	33 (33.7)	22 (40)	<0.001*
Liver	97 (16.1)	31 (31.6)	21 (38.2)	<0.001*
Peritoneum	41 (6.8)	13 (13.3)	9 (16.4)	0.002*
Adrenal gland	25 (4.2)	4 (4.1)	3 (5.5)	0.716
Skin/soft tissue	13 (2.1)	5 (5.5)	2 (3.6)	0.178
Brain	7 (1.2)	5 (5.1)	5 (9.1)	<0.001*
No. of metastatic organs	1 [0, 1]	1 [1, 2]	1 [1, 2]	<0.001*
ECOG PS				n.a.
0	357 (59.3)	0 (0.0)	0 (0.0)	
1	245 (40.7)	0 (0.0)	0 (0.0)	
2	0 (0.0)	98 (100.0)	0 (0.0)	
3	0 (0.0)	0 (0.0)	49 (89.1)	
4	0 (0.0)	0 (0.0)	6 (10.9)	

Results are presented as the median [interquartile range] or number (%).

Abbreviations: ECOG, Eastern Cooperative Oncology Group; n.a.; not applicable; NAC, neoadjuvant chemotherapy; NLR; Neutrophil–lymphocyte ratio; PS, performance status.

* *p* < 0.05

### 3.2 Response, toxicity, and survival

**FIGURE 1:**
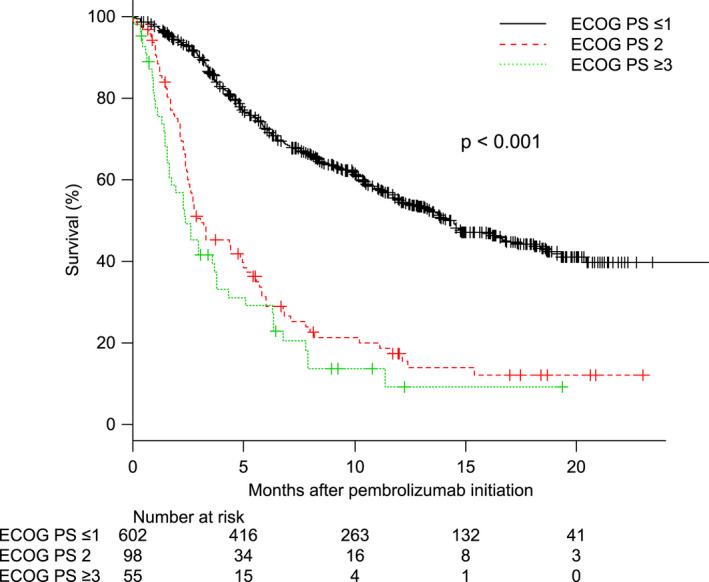
Overall survival stratified by Eastern Cooperative Oncology Group (ECOG) performance status (PS) 0–1, 2, and 3–4

Table 2 presents the response rate stratified by PS. The ORR rates in the PS 0–1, 2, and 3–4 subgroups were 29.5, 15.3, and 9.1%, respectively (*p* for trend <0.001). CR was achieved in 8.1% of patients with PS 0–1, 4.1% of patients with PS 2, and 0% of patients with PS 3–4. The estimated 1‐year PFS rates in the PS 0–1, 2, and 3–4 subgroups were 31.0, 9.8, and 8.7%, respectively (*p* for trend <0.001). Adverse events stratified by PS are presented in Table S1. The any/major (CTCAE grade ≥III) complication rates were 38.0/16.3, 34.7/15.3, and 23.6/10.9% for patients with PS 0–1, 2, and 3–4, respectively (*p* for trend =0.054/0.347). Adverse events leading to the discontinuation of pembrolizumab were observed in 9.6% of patients with PS 0–1, 6.1% of those with PS 2, and 3.6% of those with PS 3–4. Events leading to death were observed in 1.0% of patients with PS 0–1 and no patients with PS 2 or 3–4. Figure 1 presents OS curves stratified by PS subgroup. The median survival times in the PS 0–1, 2, and 3–4 subgroups were 14.3, 3.1, and 2.4 months, respectively (*p* for trend <0.001). During a median follow‐up period of 7.2 months, 274 (45.5%), 79 (80.6%), and 45 (81.8%) of patients with PS 0–1, 2, and 3–4 died, respectively. Among the deceased patients, 8 (2.9%), 6 (7.6%), and 11 patients (24.4%) with PS 0–1, 2, and 3–4, respectively, had initiated pembrolizumab within 30 days before death.

**TABLE 2:** Response outcomes stratified by performance status

	ECOG PS =0–1 n = 602	ECOG PS =2 n = 98	ECOG PS =3–4 n = 55	*p* for trend
Best response				
CR	49 (8.1)	4 (4.1)	0 (0)	
PR	129 (21.4)	11 (11.2)	5 (9.1)	
SD	149 (24.8)	13 (13.3)	6 (10.9)	
Total doses	6.00 [3.00, 12.00]	2.50 [1.00, 5.00]	2.00 [1.00, 4.00]	<0.001*
1 year PFS	31.0	9.8	8.7	<0.001*
Subsequent chemotherapies	94 (15.6)	1 (1.0)	3 (5.5)	0.003*

Results are presented as the number (%), median [interquartile range], or %.

Abbreviations: CR, complete response; ECOG, Eastern Cooperative Oncology Group; PFS progression‐free survival; PR, partial response; PS, performance status; SD, stable disease.

* *p* < 0.05.

The multivariate analysis adjusted with baseline characteristics and risk factors showed that PS ≥2 was significantly associated with both ORR (odds ratio [OR] = 0.503, 95% confidence interval [CI] = 0.287–0.881, *p* = 0.016, Table S2) and OS (hazard ratio [HR] = 2.202, 95% CI = 1.707–2.840, *p* < 0.001, Table S3).

### 3.3 Predictors of ORR and OS in the PS ≥2 subgroup

Because patients with PS 2 and 3–4 had similarly poor prognoses, the analysis to identify patients who might benefit from pembrolizumab was conducted in patients with PS ≥2. In univariate logistic regression analysis, ≥2 prior chemotherapy regimens, hemoglobin level <11 g/dl, and NLR ≥3.5 were significantly associated with ORR. In multivariate logistic regression analysis, ≥2 prior chemotherapy regimens (odds ratio [OR] = 0.11, 95% confidence interval [CI] = 0.014–0.906, *p* = 0.04), hemoglobin level <11 g/dl (OR =0 .302, 95% CI =0.106–0.857, *p* = 0.025), and NLR ≥3.5 (OR = 0.329, 95% = CI 0.120–0.903, *p* = 0.031) were independently associated with ORR (Table S4).

**FIGURE 2:**
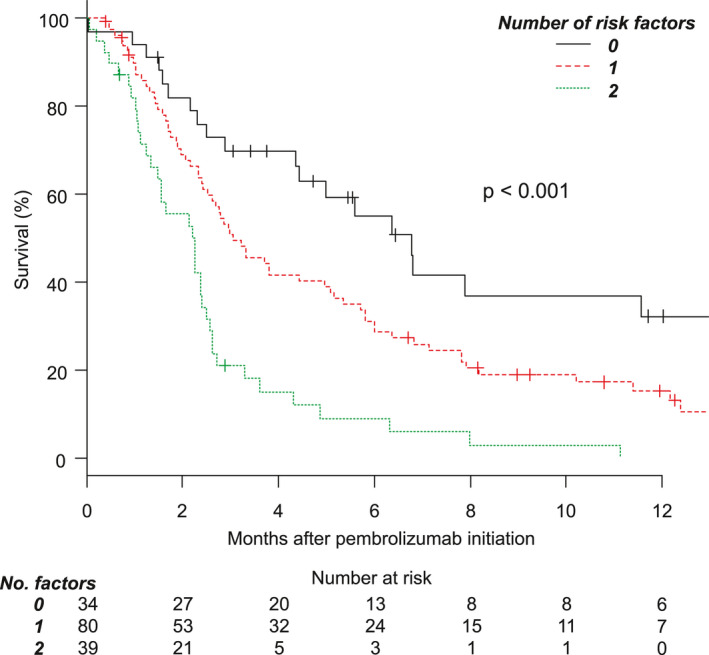
Overall survival among patients with performance status ≥2 stratified by the number of risk factors (neutrophil–lymphocyte ratio ≥3.5 and liver metastasis)

In univariate Cox regression analysis, hemoglobin level <11 g/dL, NLR ≥3.5, the presence of liver metastasis, and the number of metastatic organs ≥2 (multi‐organ metastasis) were significantly associated with prognosis. In multivariate Cox regression analysis, NLR ≥3.5 (hazard ratio [HR] = 1.894, 95% CI = 1.246–2.879, *p* = 0.003) and the presence of liver metastasis (HR = 1.982, 95% CI = 1.276–3.080, *p* = 0.002) were independently associated with prognosis (Table S5). Figure 2 presents the OS curves for patients with PS ≥2 stratified by the number of risk factors (NLR ≥3.5 and liver metastasis). The median survival time was 6.8 months for patients without either risk factor, versus 3.1 months in patients with one risk factor and 2.3 months in patients with both risk factors (p for trend <0.001). This risk classification successfully stratified the OS curve of all PS = 0–1, 2, and 3–4 subgroups into low‐, intermediate‐, and high‐risk populations (Figure S1). ORR decreased as the number of risk factors present increased as follows: 23.5% for patients with no risk factors, 13.8% for patients with one risk factor, and 2.6% for patients with both risk factors (*p* for trend = 0.007).

## 4 DISCUSSION

To the best of our knowledge, this is the first study to examine the outcomes of pembrolizumab in patients with chemorefractory advanced UC and impaired PS. In this study, patients with PS 2 and 3–4 displayed worse ORR and OS than patients with PS 0–1, whereas the safety profile was comparable among these subgroups. In particular, this study included 55 patients with PS 3–4. The survival curves for the PS 2 and 3–4 subgroups were comparable, but almost 20% of patients with PS 3–4 died within 30 days after the initiation of pembrolizumab therapy. Hemoglobin levels, NLR, the number of prior chemotherapy regimens, and the presence of liver metastasis were significantly associated with ORR and OS for patients with PS 2–4. Survival time analysis illustrated patients with NLR <3.5 and no liver metastases, regardless of the PS, can benefit from pembrolizumab treatment.

In this study, PS ≥2 was significantly associated with shorter OS and lower ORR than PS ≤1. In the KEYNOTE‐045 trial, the median OS was 10.3 months, and the ORR was 21.1%,^1^ which were comparable to our results in patients with PS ≤1. The trial also revealed the survival benefit of pembrolizumab over salvage chemotherapy in patients with PS 2. However, this study enrolled only six patients with PS 2. Moreover, patients with PS 2 and at least one Bellmunt risk factor (i.e., hemoglobin concentration <10 g/dl, presence of liver metastases, and receipt of the last dose of chemotherapy <3 months before enrollment) were excluded. Thus, patients with PS 2 in the KEYNOTE‐045 trial differ significantly from the actual population of patients with UC and PS 2. Four retrospective studies have assessed efficacy of ICIs in patients with UC and PS ≥2, including any ICI^8^ in one study and atezolizumab in three studies.^9^, ^10^, ^11^ Khaki et al. reported a median OS of 8.2 months and ORR of 23% among patients with PS ≥2 and who received an ICI in the second‐line setting or later, which were comparable to the outcomes for patients with PS ≤1.^8^ Conversely, a study of atezolizumab therapy in patients with advanced UC or non‐UC (SAUL study) reported a median OS of 2.3 months and ORR of 5% in patients with PS 2.^9^ The other two studies did not focus on patients with PS ≥2, and the reported ORR for the entire cohorts ranged from 17 to 21%.^10^, ^11^ These widely divergent outcomes might be attributable to differences in patients’ backgrounds such as the number of risk factors. Of note, our study included more than 50 patients with PS 3–4. The survival curves were similar between patients with PS 3–4 and 2, but ORR and the proportion of the patients who died within 30 days after the initiation of pembrolizumab were worse in the former subgroup. Although the use of pembrolizumab for patients with PS 4 was specially selected cases, one out of six patients with PS 4 achieved PR. It should be noted that the definition of PS does not consider comorbidities or cancer stage separately, and it is also difficult to determine whether the cause of PS decline is cancer alone or the effect of comorbidities. This indicates the existence of a group of patients with PS 3–4 who may benefit from pembrolizumab therapy even though its efficacy generally declining with worsening PS.

Because patients with PS ≥2 represent a heterogeneous population, there is a need to predict the outcomes of pembrolizumab treatment in these patients. In such patients, survival outcomes were clearly influenced by the number of risk factors (NLR ≥3.5 and liver metastasis). Interestingly, the median OS was similar for the PS 2 and 3–4 subgroups when stratified by the number of risk factors. In addition, the OS for patients with PS ≥2 was worse than that of PS 0–1 in each risk group. These results suggest that PS have a strong association with prognosis, and NLR and liver metastasis influence prognosis regardless of PS. Patients with PS 2 and 3–4 without risk factors may benefit from pembrolizumab even though OS in these patients is worse than that in patients with PS 0–1. Patients with both risk factors are likely to die within several months after the start of treatment; thus, the effect of pembrolizumab on survival would be minimal. Our data concerning the real‐world outcomes of pembrolizumab in patients with impaired PS will certainly aid decision making concerning the administration of pembrolizumab.

This study identified that hemoglobin levels <11 g/dl, NLR ≥3.5, and ≥2 prior chemotherapy regimens were independently associated with ORR, whereas NLR ≥3.5 and the presence of liver metastasis were associated with prognosis. The site of metastasis, hemoglobin levels, and NLR were independent prognostic factors in the entire cohort, as reported elsewhere.^6^ This study illustrated that these factors can be predictors of ORR and prognosis in patients with PS ≥2. Hemoglobin levels and liver metastasis have been proposed as independent predictors of PFS and OS for patients with platinum‐refractory advanced UC.^2^, ^12^ In addition, NLR displayed the strongest association with ORR and OS in this study. NLR is considered to reflect systemic inflammation caused by cancer.^13^ High NLRs have been reported to be significant discriminators of the response to chemotherapy or survival in various types of cancers,^13^ including UC.^14^, ^15^, ^16^, ^17^ In this study, NLR ≥3.5 was adopted as the optimal cutoff because a cutoff of 3.0 resulted in an extremely small proportion of patients with PS ≥2. The interval since previous chemotherapy was not significantly associated with ORR and OS. Instead, the number of prior chemotherapy regimens was significantly associated with ORR. This might be because this cohort represents the initial experience with pembrolizumab. A certain number of patients had received multiple chemotherapy regimens or waited a long period prior to the approval of pembrolizumab in December 2017.

In our cohort, the toxicity rate did not significantly differ among the patients according to PS, although this finding might have been influenced by the short duration of treatment and survival in the poor PS subgroup. This tendency was observed in the SAUL study, which reported that 53% of all patients and 35% of those with PS 2 who received atezolizumab experienced treatment‐related complications.^9^ In this respect, immunotherapy is easier to use in patients with impaired PS than chemotherapy, which is more likely to cause severe adverse events in such patients. In fact, the use of immunotherapy use near the end of life is increasing. In the US, the rate of ICI initiation within the last 60 days of life in patients with UC increased from 1.0% in the fourth quarter of 2015 to 23% in the fourth quarter of 2017.^5^ Although ICIs might be safer than traditional chemotherapy in patients with impaired PS, we must note that more than 10% of patients with PS ≥2 experienced grade III or worse adverse events in our study. Moreover, ICI use near the end of life is associated with financial difficulties and lower hospice enrollment.^8^, ^18^ Given the ORR of 2.6% and mortality rate of 78.9% within 3 months in patients with PS ≥2 and the two aforementioned risk factors (NLR ≥3.5 and liver metastasis), the initiation of pembrolizumab for these patients should be carefully considered with detailed discussion.

This study had several limitations. First, because of its retrospective nature, patient characteristics among patients with PS ≤1, 2, or ≥3 were largely different. Although the effect of PS on survival and ORR remained significant in multivariate analysis, potential confounders such as total tumor volume still exist. The indication for treatment, timing of evaluation, and use of pre‐ or post‐treatment regimens also varied. The evaluation of toxicity was not centralized and the person in charge in each institution reported the toxicity when ICI was the most likely cause of the symptom. It was particularly difficult to distinguish ICI’s toxicity from the symptoms due to cancer progression in patients with impaired PS. Second, the ECOG‐PS assessment is inherently subjective and does not take age, comorbidities, or stage of a cancer into account. Moreover, there are discrepancies between patients’ and clinicians’ evaluation of ECOG‐PS.^19^ Alternative tools that can objectively assess patients’ physical status and its prognostic value should be further studied.^20^ Third, this study was a single‐arm trial that lacked a control or traditional chemotherapy group. Because PS ≥2 is usually a contraindication for standard chemotherapy, it is difficult to compare outcomes. Patients with PS 2, such as those who lack our proposed risk factors, should be enrolled in prospective controlled trials. In addition, it is unclear whether pembrolizumab has a positive benefit on survival versus best supportive care for patients with PS ≥2. Given the extremely short OS in patients with several risk factors, pembrolizumab may have little or no prognostic benefit for these patients. Moreover, the effects of this ICI on the quality of life or financial burden of patients should be further investigated. Fourth, the potential laboratory markers, such as platelet‐to‐lymphocyte ratio or LDH,^21^ were not collected in this retrospective study. It should be further investigated which laboratory markers are more predictive. We have newly started the prospective cohort study which includes these variables. Finally, this study did not examine proposed biomarkers such as PD‐L1 expression, the tumor mutational burden, or the tumor microenvironment.^22^ These biomarkers may be introduced as decision‐making tools in the future, but they have not been widely accepted in real practice. Until the utility of these biomarkers is fully validated, PS should be utilized for decision making.

In conclusion, patients with PS ≥2 exhibited worse ORR and OS than those with PS ≤1 despite their comparable safety profiles. The survival curves were similar between patients with PS 2 and 3–4. High NLR (≥3.5) and the presence of liver metastasis were significant risk factors for worse OS in patients with PS ≥2.

## CONFLICT OF INTEREST

All authors declare there is no conflict of interest.

## ETHICAL STATEMENT

This multi‐institutional study was approved by the Kyoto University review board (R1783, approved in June 2018) followed by the review boards of all 59 participating institutions.

## Supporting information

Fig S1

Table S1

Table S2

Table S3

Table S4

Table S5

## Data Availability

The data that support the findings of this study are available from the corresponding author upon reasonable request.

## 1 Study institutes

Osaka City University, Akita University, Hirosaki University, National Cancer Center Hospital, Hamamatsu University School of Medicine, Yamagata University, Kyoto University, University of Tsukuba, Nara Medical University, Shizuoka General Hospital, University of the Ryukyus, Iwate Medical University, Oita University, Hiroshima University, Shimane University, Kansai Medical University, Osaka University, Kagawa University, University of Yamanashi, Japanese Red Cross Wakayama Medical Center, Kyoto Prefectural University of Medicine, Kobe City Nishi‐Kobe Medical Center, Japanese Red Cross Osaka Hospital, Nagoya University, Harasanshin Hospital, Hokkaido University, Japanese Red Cross Otsu Hospital, Kagoshima University, Kyushu University, Shikoku Cancer Center, Tenri Hospital, Hakodate Goryoukaku Hospital, Kitasato University, Kyoto Katsura Hospital, National Hospital Organization Kyoto Medical Center, Kumamoto University, National Hospital Organization Himeji Medical Center, Tazuke Kofukai Medical Research Institute Kitano Hospital, Toyooka Hospital, Hokkaido Cancer Center, University of Miyazaki, Hitachi General Hospital, The Jikei University Kashiwa Hospital, Shimada Municipal Hospital, Mie University, Yamaguchi University, Ibaraki Prefectural Central Hospital, Kyoto City Hospital, Kochi University, Ijinkai Takeda General Hospital, University of Toyama, Otsu City Hospital, Sapporo Medical University, The Jikei University Hospital, Kansai Electric Power Hospital, Kurume University, Hyogo College of Medicine, Hirakata Kohsai Hospital, Rakuwakai Otowa Hospital.

## References

[1] Bellmunt J , de Wit R , Vaughn DJ , et al. Pembrolizumab as second‐line therapy for advanced urothelial carcinoma. N Engl J Med. 2017;376(11):1015‐1026. 28212060 10.1056/NEJMoa1613683 PMC5635424

[2] Bellmunt J , Choueiri TK , Fougeray R , et al. Prognostic factors in patients with advanced transitional cell carcinoma of the urothelial tract experiencing treatment failure with platinum‐containing regimens. J Clin Oncol. 2010;28(11):1850‐1855. 20231682 10.1200/JCO.2009.25.4599

[3] Bajorin DF , Dodd PM , Mazumdar M , et al. Long‐term survival in metastatic transitional‐cell carcinoma and prognostic factors predicting outcome of therapy. J Clin Oncol. 1999;17(10):3173‐3181. 10506615 10.1200/JCO.1999.17.10.3173

[4] Caires‐Lima R , Cayres K , Protasio B , et al. Palliative chemotherapy outcomes in patients with ECOG‐PS higher than 1. Ecancermedicalscience. 2018;12:831. 29743951 10.3332/ecancer.2018.831 PMC5931814

[5] Parikh RB , Galsky MD , Gyawali B , et al. Trends in checkpoint inhibitor therapy for advanced urothelial cell carcinoma at the end of life: insights from real‐world practice. Oncologist. 2019;24(6):e397‐e399. 30944183 10.1634/theoncologist.2019-0039 PMC6656487

[6] Kobayashi T , Ito K , Kojima T , et al. Risk stratification for the prognosis of patients with chemoresistant urothelial cancer treated with pembrolizumab. Cancer Sci. 2021;112(2):760‐773. 33283385 10.1111/cas.14762 PMC7893997

[7] Kanda Y . Investigation of the freely available easy‐to‐use software ‘EZR’ for medical statistics. Bone Marrow Transplant. 2013;48(3):452‐458. 23208313 10.1038/bmt.2012.244 PMC3590441

[8] Khaki AR , Li A , Diamantopoulos LN , et al. Impact of performance status on treatment outcomes: a real‐world study of advanced urothelial cancer treated with immune checkpoint inhibitors. Cancer. 2020;126(6):1208‐1216. 31829450 10.1002/cncr.32645 PMC7050422

[9] Sternberg CN , Loriot Y , James N , et al. Primary results from SAUL, a multinational single‐arm safety study of atezolizumab therapy for locally advanced or metastatic urothelial or nonurothelial carcinoma of the urinary tract. Eur Urol. 2019;76(1):73‐81. 30910346 10.1016/j.eururo.2019.03.015

[10] Barata PC , Gopalakrishnan D , Koshkin VS , et al. Atezolizumab in metastatic urothelial carcinoma outside clinical trials: focus on efficacy, safety, and response to subsequent therapies. Target Oncol. 2018;13(3):353‐361. 29623487 10.1007/s11523-018-0561-6

[11] Pal SK , Hoffman‐Censits J , Zheng H , Kaiser C , Tayama D , Bellmunt J . Atezolizumab in platinum‐treated locally advanced or metastatic urothelial carcinoma: clinical experience from an expanded access study in the United States. Eur Urol. 2018;73(5):800‐806. 29478735 10.1016/j.eururo.2018.02.010

[12] Pond GR , Agarwal N , Bellmunt J , et al. A nomogram including baseline prognostic factors to estimate the activity of second‐line therapy for advanced urothelial carcinoma. BJU Int. 2014;113(5b):E137‐E143. 24219029 10.1111/bju.12564

[13] Templeton AJ , McNamara MG , Seruga B , et al. Prognostic role of neutrophil‐to‐lymphocyte ratio in solid tumors: a systematic review and meta‐analysis. J Natl Cancer Inst. 2014;106(6):dju124. 24875653 10.1093/jnci/dju124

[14] Tan YG , Eu EWC , Huang HH , Lau WKO . High neutrophil‐to‐lymphocyte ratio predicts worse overall survival in patients with advanced/metastatic urothelial bladder cancer. Int J Urol. 2018;25(3):232‐238. 29094397 10.1111/iju.13480

[15] Taguchi S , Nakagawa T , Matsumoto A , et al. Pretreatment neutrophil‐to‐lymphocyte ratio as an independent predictor of survival in patients with metastatic urothelial carcinoma: a multi‐institutional study. Int J Urol. 2015;22(7):638‐643. 25903328 10.1111/iju.12766

[16] Yoshida T , Kobayashi T , Kawaura T , et al. Development and external validation of a preoperative nomogram for predicting pathological locally advanced disease of clinically localized upper urinary tract carcinoma. Cancer Med. 2020;9(11):3733‐3741. 32253820 10.1002/cam4.2988 PMC7286474

[17] Ogihara K , Kikuchi E , Shigeta K , et al. The pretreatment neutrophil‐to‐lymphocyte ratio is a novel biomarker for predicting clinical responses to pembrolizumab in platinum‐resistant metastatic urothelial carcinoma patients. Urol Oncol. 2020;38(6):602.e1‐602.e10. 10.1016/j.urolonc.2020.02.005 32139290

[18] Glisch C , Saeidzadeh S , Snyders T , Gilbertson‐White S , Hagiwara Y , Lyckholm L . Immune checkpoint inhibitor use near the end of life: a single‐center retrospective study. J Palliat Med. 2020;23(7):977‐979 31702481 10.1089/jpm.2019.0383

[19] Bergerot CD , Philip EJ , Bergerot PG , et al. Discrepancies between genitourinary cancer patients’ and clinicians’ characterization of the Eastern Cooperative Oncology Group performance status. Cancer. 2021;127(3):354‐358. 33007114 10.1002/cncr.33238

[20] Scott JM , Stene G , Edvardsen E , Jones LW . Performance status in cancer: not broken, but time for an upgrade? J Clin Oncol. 2020;38(25):2824‐2829. 32584631 10.1200/JCO.20.00721 PMC7460152

[21] Russo A , Russano M , Franchina T , et al. Neutrophil‐to‐lymphocyte ratio (NLR), platelet‐to‐lymphocyte ratio (PLR), and outcomes with nivolumab in pretreated non‐small cell lung cancer (NSCLC): a large retrospective multicenter study. Adv Ther. 2020;37(3):1145‐1155. 32002809 10.1007/s12325-020-01229-w

[22] Powles T , Kockx M , Rodriguez‐Vida A , et al. Clinical efficacy and biomarker analysis of neoadjuvant atezolizumab in operable urothelial carcinoma in the ABACUS trial. Nat Med. 2019;25(11):1706‐1714. 31686036 10.1038/s41591-019-0628-7

