# Supplementary material for: Pembrolizumab for treating advanced urothelial carcinoma in patients with impaired performance status: Analysis of a Japanese nationwide cohort
Source: Cancer Med. 2021 May 1;10(10):3188–96. doi: 10.1002/cam4.3863 (PMC8124127; doi:10.1002/cam4.3863)
Supplement: Supplementary file 1 — Fig S1 [file CAM4-10-3188-s005.pdf]

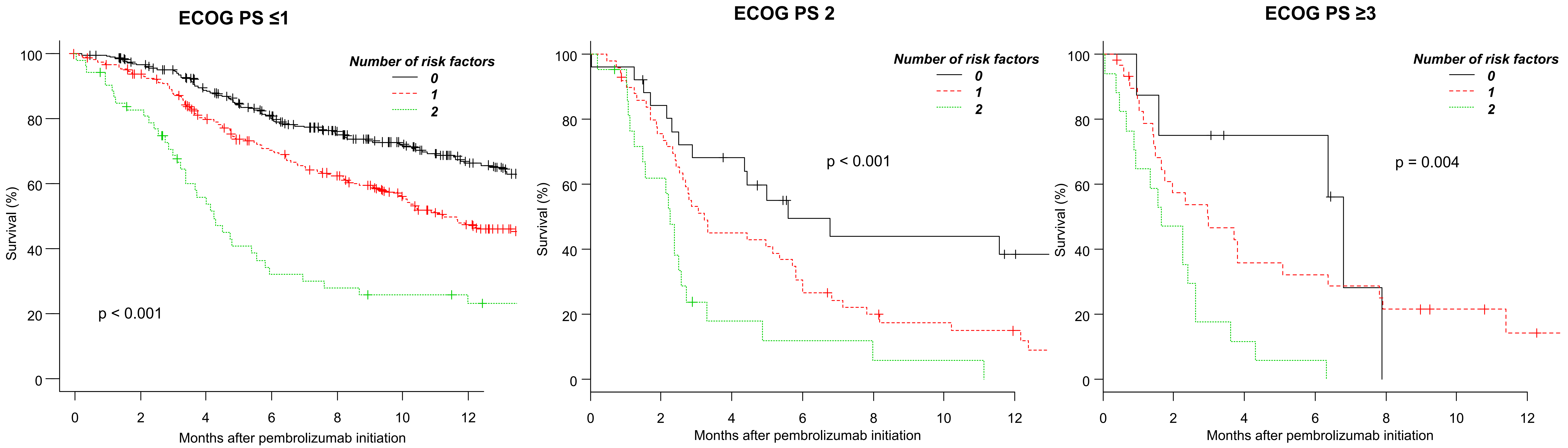

ECOG PS ≤1: Number at risk

No. factors							
0	299	281	246	210	172	144	111
1	243	222	181	151	130	105	80
2	53	42	26	15	13	11	10

ECOG PS 2: Number at risk

No. factors							
0	26	21	16	9	8	8	6
1	50	37	22	15	9	7	5
2	22	13	3	2	1	1	0

ECOG PS ≥3: Number at risk

No. factors							
0	8	6	4	4	0	0	0
1	30	16	10	9	6	4	2
2	17	8	2	1	0	0	0

Supplementary Figure 1. Overall survival among patients with Eastern Cooperative Oncology Group (ECOG) performance status (PS) ≤1, 2 and PS ≥ 3 stratified by number of risk factors (neutrophil-lymphocyte ratio ≥ 3.5 and liver metastasis).
