# Supplementary material for: Pembrolizumab for treating advanced urothelial carcinoma in patients with impaired performance status: Analysis of a Japanese nationwide cohort
Source: Cancer Med. 2021 May 1;10(10):3188–96. doi: 10.1002/cam4.3863 (PMC8124127; doi:10.1002/cam4.3863)
Supplement: Supplementary file 2 — Table S1 [file CAM4-10-3188-s001.docx]

**Supp. Table 1.** Complications stratified by performance status

|  | ECOG PS = 0–1 n = 602 | | ECOG PS = 2 n = 98 | | ECOG PS = 3–4 n = 55 | |
| --- | --- | --- | --- | --- | --- | --- |
|  | All | Major (grade ≥ III) | All | Major (grade ≥ III) | All | Major　 (grade ≥ III) |
| Any event | 229(38) | 98(16.3) | 34(34.7) | 15(15.3) | 13(23.6) | 6(10.9) |
| Event leading to discontinuation of pembrolizumab | 58(9.6) | 33(5.5) | 6(6.1) | 4(4.1) | 2(3.6) | 2(3.6) |
| Event leading to death | 6(1) | 6(1) | 0(0) | 0(0) | 0(0) | 0(0) |
| Skin disorder | 56(9.3) | 7(1.2) | 4(4.1) | 1(1) | 1(1.8) | 0(0) |
| Endocrine disorder | 56(9.3) | 13(2.2) | 6(6.1) | 2(2) | 0(0) | 0(0) |
| Gastrointestinal disorder/diarrhea | 47(7.8) | 14(2.3) | 2(2) | 1(1) | 3(5.5) | 1(1.8) |
| Liver injury/hepatitis | 34(5.6) | 12(2) | 5(5.1) | 1(1) | 2(3.6) | 2(3.6) |
| Lung injury/interstitial pneumonia | 33(5.5) | 23(3.8) | 8(8.2) | 4(4.1) | 0(0) | 0(0) |
| General fatigue | 21(3.5) | 4(0.7) | 9(9.2) | 5(5.1) | 3(5.5) | 2(3.6) |
| Infection | 18(3.0) | 13(2.2) | 1(1) | 1(1) | 2(3.6) | 1(1.8) |
| Kidney injury | 13(2.2) | 11(1.8) | 2(2) | 1(1) | 3(5.5) | 1(1.8) |
| Neurological disorder | 13(2.2) | 1(0.2) | 2(2) | 1(1) | 2(3.6) | 0(0) |
| Myelosuppression | 13(2.2) | 3(0.5) | 1(1) | 0(0) | 0(0) | 0(0) |
| Infusion reaction | 5(0.8) | 0(0) | 0(0) | 0(0) | 0(0) | 0(0) |
| Arthritis | 3(0.5) | 1(0.2) | 0(0) | 0(0) | 0(0) | 0(0) |
| Stomatitis | 2(0.3) | 0(0) | 0(0) | 0(0) | 0(0) | 0(0) |
| Diabetes mellitus | 2(0.3) | 1(0.2) | 0(0) | 0(0) | 0(0) | 0(0) |
| Pancreatitis | 2(0.3) | 2(0.3) | 0(0) | 0(0) | 0(0) | 0(0) |
| Uveitis | 1(0.2) | 0(0) | 0(0) | 0(0) | 0(0) | 0(0) |
| CK elevation | 1(0.2) | 1(0.2) | 0(0) | 0(0) | 0(0) | 0(0) |
| Pleural effusion | 1(0.2) | 1(0.2) | 0(0) | 0(0) | 0(0) | 0(0) |
| Edema | 1(0.2) | 0(0) | 2(2) | 0(0) | 1(1.8) | 0(0) |
| Polymyositis/Dermatitis | 1(0.2) | 1(0.2) | 0(0) | 0(0) | 0(0) | 0(0) |
| Heart failure | 1(0.2) | 1(0.2) | 0(0) | 0(0) | 0(0) | 0(0) |
| Meningoencephalitis | 0(0) | 0(0) | 1(1) | 1(1) | 0(0) | 0(0) |
| Thrombocytopenia | 1(0.2) | 1(0.2) | 0(0) | 0(0) | 0(0) | 0(0) |
| Sialadenitis | 1(0.2) | 1(0.2) | 0(0) | 0(0) | 0(0) | 0(0) |
| Hypercalcemia | 0(0) | 0(0) | 0(0) | 0(0) | 1(1.8) | 1(1.8) |

Results are presented as the number (%). Abbreviations: ECOG, Eastern Cooperative Oncology Group; PS, performance status.
