# Supplementary material for: Pembrolizumab for treating advanced urothelial carcinoma in patients with impaired performance status: Analysis of a Japanese nationwide cohort
Source: Cancer Med. 2021 May 1;10(10):3188–96. doi: 10.1002/cam4.3863 (PMC8124127; doi:10.1002/cam4.3863)
Supplement: Supplementary file 3 — Table S2 [file CAM4-10-3188-s002.docx]

**Supp. Table 2.** Univariate and multivariate logistic regression analyses of objective response (complete response or partial response) among 755 patients

|  | Univariate | | | | Multivariate | | | |  |
| --- | --- | --- | --- | --- | --- | --- | --- | --- | --- |
|  | OR | 95% CI lower | 95% CI upper | P value | OR | 95% CI lower | 95% CI upper | P value | |
| Age at initiation, year | 1.000 | 0.984 | 1.020 | 0.820 |  |  |  |  | |
| Sex, male | 1.040 | 0.712 | 1.520 | 0.842 |  |  |  |  | |
| Current or past smoker | 0.820 | 0.586 | 1.150 | 0.247 |  |  |  |  | |
| Primary site of UC, bladder | 1.020 | 0.739 | 1.410 | 0.892 |  |  |  |  | |
| Variant histology | 1.230 | 0.717 | 2.110 | 0.452 |  |  |  |  | |
| Prior cystectomy or nephroureterectomy | 1.520 | 1.090 | 2.120 | 0.014* | 1.310 | 0.913 | 1.870 | 0.144 | |
| Number of prior chemotherapy ≥ 2 | 0.127 | 0.016 | 0.981 | 0.048* | 0.871 | 0.576 | 1.320 | 0.511 | |
| < 90 days after prior chemotherapy | 0.767 | 0.553 | 1.060 | 0.111 |  |  |  |  | |
| Hemoglobin, g/dL | 1.140 | 1.050 | 1.240 | 0.003* | 1.050 | 0.947 | 1.170 | 0.336 | |
| Albumin, g/dL | 1.510 | 1.140 | 2.010 | 0.004* | 0.922 | 0.627 | 1.350 | 0.678 | |
| NLR | 0.927 | 0.881 | 0.975 | 0.003* | 0.951 | 0.902 | 1.000 | 0.067 | |
| Lymph node metastasis | 1.030 | 0.729 | 1.460 | 0.861 |  |  |  |  | |
| Visceral metastasis |  |  |  |  |  |  |  |  | |
| Lung | 0.706 | 0.503 | 0.991 | 0.044* | 0.700 | 0.407 | 1.200 | 0.196 | |
| Bone | 0.310 | 0.184 | 0.522 | <0.001* | 0.339 | 0.166 | 0.693 | 0.003* | |
| Liver | 0.450 | 0.279 | 0.725 | 0.001* | 0.632 | 0.323 | 1.240 | 0.180 | |
| Peritoneum | 0.954 | 0.528 | 1.720 | 0.876 |  |  |  |  | |
| Adrenal gland | 0.780 | 0.332 | 1.830 | 0.568 |  |  |  |  | |
| Skin/soft tissue | 1.210 | 0.459 | 3.200 | 0.698 |  |  |  |  | |
| Brain | 0.597 | 0.170 | 2.100 | 0.421 |  |  |  |  | |
| No. of metastatic organs | 0.626 | 0.513 | 0.764 | <0.001* | 1.050 | 0.688 | 1.600 | 0.821 | |
| ECOG PS ≥ 2 | 0.358 | 0.217 | 0.592 | <0.001* | 0.503 | 0.287 | 0.881 | 0.016* | |

Abbreviations: CI, confidence interval; ECOG, Eastern Cooperative Oncology Group; NLR, neutrophil-lymphocyte ratio; OR, odds ratio; PS, performance status; UC, urothelial cancer. *P < 0.05.
