# Supplementary material for: Pembrolizumab for treating advanced urothelial carcinoma in patients with impaired performance status: Analysis of a Japanese nationwide cohort
Source: Cancer Med. 2021 May 1;10(10):3188–96. doi: 10.1002/cam4.3863 (PMC8124127; doi:10.1002/cam4.3863)
Supplement: Supplementary file 4 — Table S3 [file CAM4-10-3188-s004.docx]

**Supp. Table 3.** Univariate and multivariate Cox regression analyses of overall survival among 755 patients

|  | Univariate | | | | | Multivariate | | | |  |
| --- | --- | --- | --- | --- | --- | --- | --- | --- | --- | --- |
|  | HR | 95% CI lower | 95% CI upper | P value | HR | | 95% CI lower | 95% CI upper | P value | |
| Age at initiation, year | 1.000 | 0.990 | 1.011 | 0.983 |  | |  |  |  | |
| Sex, male | 0.953 | 0.760 | 1.193 | 0.672 |  | |  |  |  | |
| Current or past smoker | 1.126 | 0.915 | 1.384 | 0.262 |  | |  |  |  | |
| Primary site of UC, bladder | 0.899 | 0.739 | 1.095 | 0.292 |  | |  |  |  | |
| Variant histology | 0.814 | 0.567 | 1.169 | 0.265 |  | |  |  |  | |
| Prior cystectomy or nephroureterectomy | 0.700 | 0.575 | 0.853 | <0.001* | 0.800 | | 0.650 | 0.983 | 0.034* | |
| Number of prior chemotherapy ≥ 2 | 1.137 | 0.911 | 1.418 | 0.256 |  | |  |  |  | |
| < 90 days after prior chemotherapy | 1.439 | 1.181 | 1.754 | <0.001* | 1.073 | | 0.866 | 1.329 | 0.522 | |
| Hemoglobin, g/dL | 0.819 | 0.778 | 0.862 | <0.001* | 0.948 | | 0.885 | 1.015 | 0.125 | |
| Albumin, g/dL | 0.449 | 0.384 | 0.523 | <0.001* | 0.698 | | 0.564 | 0.864 | 0.001* | |
| NLR | 1.033 | 1.023 | 1.043 | <0.001* | 1.015 | | 1.000 | 1.029 | 0.041* | |
| Lymph node metastasis | 1.235 | 0.994 | 1.534 | 0.056 |  | |  |  |  | |
| Visceral metastasis |  |  |  |  |  | |  |  |  | |
| Lung | 1.322 | 1.084 | 1.612 | 0.006* | 0.833 | | 0.445 | 1.558 | 0.567 | |
| Bone | 2.293 | 1.835 | 2.864 | <0.001* | 1.036 | | 0.544 | 1.972 | 0.915 | |
| Liver | 2.475 | 1.983 | 3.090 | <0.001* | 1.286 | | 0.693 | 2.388 | 0.425 | |
| Peritoneum | 1.562 | 1.138 | 2.144 | 0.006* | 0.855 | | 0.416 | 1.755 | 0.669 | |
| Adrenal gland | 1.675 | 10.89 | 2.576 | 0.019* | 0.832 | | 0.385 | 1.797 | 0.639 | |
| Skin/soft tissue | 1.398 | 0.787 | 2.484 | 0.254 |  | |  |  |  | |
| Brain | 2.663 | 1.588 | 4.466 | <0.001* | 1.278 | | 0.577 | 2.829 | 0.546 | |
| No. of metastatic organs | 1.701 | 1.537 | 1.882 | <0.001* | 1.514 | | 0.842 | 2.725 | 0.166 | |
| ECOG PS ≥ 2 | 3.650 | 2.939 | 4.534 | <0.001* | 2.202 | | 1.707 | 2.840 | <0.001* | |

Abbreviations: CI, confidence interval; ECOG, Eastern Cooperative Oncology Group; NLR, neutrophil-lymphocyte ratio; OR, odds ratio; PS, performance status; UC, urothelial cancer. *P < 0.05.
