# Supplementary material for: Pembrolizumab for treating advanced urothelial carcinoma in patients with impaired performance status: Analysis of a Japanese nationwide cohort
Source: Cancer Med. 2021 May 1;10(10):3188–96. doi: 10.1002/cam4.3863 (PMC8124127; doi:10.1002/cam4.3863)
Supplement: Supplementary file 5 — Table S4 [file CAM4-10-3188-s006.docx]

**Supp. Table 4.** Univariate and multivariate logistic regression analyses of objective response (complete response or partial response) among 153 patients with impaired performance status (≥2)

|  | No of patients | Univariate | | | | | Multivariate | | | | |
| --- | --- | --- | --- | --- | --- | --- | --- | --- | --- | --- | --- |
|  | n = 153 | OR | 95% CI lower | 95% CI upper | P value | OR | | 95% CI lower | 95% CI upper | P value |  |
| Age at initiation, year |  | 0.970 | 0.926 | 1.020 | 0.201 |  | |  |  |  |  |
| Sex, male | 112 | 1.540 | 0.484 | 4.910 | 0.464 |  | |  |  |  |  |
| Current or past smoker | 90 | 0.923 | 0.352 | 2.420 | 0.871 |  | |  |  |  |  |
| Primary site of UC, bladder | 82 | 0.672 | 0.261 | 1.730 | 0.410 |  | |  |  |  |  |
| Variant histology | 18 | 0.813 | 0.172 | 3.830 | 0.793 |  | |  |  |  |  |
| Prior cystectomy or nephroureterectomy | 77 | 1.240 | 0.483 | 3.190 | 0.654 |  | |  |  |  |  |
| Number of prior chemotherapy ≥ 2 | 40 | 0.127 | 0.016 | 0.981 | 0.048* | 0.113 | | 0.014 | 0.906 | 0.040* |  |
| < 90 days after prior chemotherapy | 84 | 1.000 | 0.391 | 2.580 | 0.992 |  | |  |  |  |  |
| Hemoglobin < 11 g/dL | 118 | 0.297 | 0.112 | 0.791 | 0.015* | 0.302 | | 0.106 | 0.857 | 0.025* |  |
| Albumin < 2.5 g/dL | 25 | 1.130 | 0.303 | 4.210 | 0.857 |  | |  |  |  |  |
| NLR ≥ 3.5 | 106 | 0.304 | 1.116 | 0.793 | 0.015* | 0.329 | | 0.120 | 0.903 | 0.031* |  |
| Liver metastasis | 51 | 0.446 | 0.172 | 1.150 | 0.096 |  | |  |  |  |  |
| Visceral metastasis |  |  |  |  |  |  | |  |  |  |  |
| Lung | 65 | 0.696 | 0.261 | 1.860 | 0.469 |  | |  |  |  |  |
| Bone | 55 | 0.553 | 0.190 | 1.610 | 0.279 |  | |  |  |  |  |
| Liver | 52 | 0.443 | 0.140 | 1.400 | 0.165 |  | |  |  |  |  |
| Peritoneum | 22 | 2.270 | 0.733 | 7.060 | 0.155 |  | |  |  |  |  |
| Adrenal gland | 7 | 0 | 0 | Inf | 0.992 |  | |  |  |  |  |
| Skin/soft tissue | 7 | 0 | 0 | Inf | 0.992 |  | |  |  |  |  |
| Brain | 10 | 1.740 | 0.341 | 8.830 | 0.506 |  | |  |  |  |  |
| No. of metastatic organs ≥2 | 70 | 0.763 | 0.293 | 1.99 | 0.581 |  | |  |  |  |  |
| ECOG PS≥3 (vs PS=2) | 55 | 0.553 | 0.190 | 1.610 | 0.279 |  | |  |  |  |  |

Abbreviations: CI, confidence interval; ECOG, Eastern Cooperative Oncology Group; NLR, neutrophil-lymphocyte ratio; OR, odds ratio; PS, performance status; UC, urothelial cancer. *P < 0.05.
