# Supplementary material for: Pembrolizumab for treating advanced urothelial carcinoma in patients with impaired performance status: Analysis of a Japanese nationwide cohort
Source: Cancer Med. 2021 May 1;10(10):3188–96. doi: 10.1002/cam4.3863 (PMC8124127; doi:10.1002/cam4.3863)
Supplement: Supplementary file 6 — Table S5 [file CAM4-10-3188-s003.docx]

**Supp. Table 5.** Univariate and multivariate Cox regression analyses of overall survival among 153 patients with impaired performance status (≥2)

|  | no. patients | Univariate | | | | | Multivariate | | | | |
| --- | --- | --- | --- | --- | --- | --- | --- | --- | --- | --- | --- |
|  | n = 153 | HR | 95% CI lower | 95% CI upper | P value | HR | | 95% CI lower | 95% CI upper | P value |  |
| Age at initiation, year |  | 1.015 | 0.996 | 1.034 | 0.131 |  | |  |  |  |  |
| Sex, male | 112 | 1.249 | 0.834 | 1.871 | 0.281 |  | |  |  |  |  |
| Current or past smoker | 90 | 1.220 | 0.837 | 1.778 | 0.302 |  | |  |  |  |  |
| Primary site of UC, bladder | 82 | 0.716 | 0.502 | 1.020 | 0.064 |  | |  |  |  |  |
| Variant histology | 18 | 0.888 | 0.509 | 1.551 | 0.676 |  | |  |  |  |  |
| Prior cystectomy or nephroureterectomy | 77 | 0.985 | 0.692 | 1.404 | 0.935 |  | |  |  |  |  |
| Number of prior chemotherapy ≥ 2 | 40 | 1.438 | 0.973 | 2.126 | 0.069 |  | |  |  |  |  |
| < 90 days after prior chemotherapy | 84 | 1.149 | 0.804 | 1.642 | 0.446 |  | |  |  |  |  |
| Hemoglobin < 11 g/dL | 118 | 1.630 | 1.045 | 2.544 | 0.031* | 1.468 | | 0.937 | 2.299 | 0.094 |  |
| Albumin < 2.5 g/dL | 25 | 1.306 | 0.827 | 2.063 | 0.252 |  | |  |  |  |  |
| NLR ≥ 3.5 | 106 | 1.931 | 1.275 | 2.924 | 0.002* | 1.894 | | 1.246 | 2.879 | 0.003* |  |
| Lymph node metastasis | 51 | 1.380 | 0.935 | 2.036 | 0.105 |  | |  |  |  |  |
| Visceral metastasis |  |  |  |  |  |  | |  |  |  |  |
| Lung | 65 | 1.278 | 0.895 | 1.824 | 0.177 |  | |  |  |  |  |
| Bone | 55 | 1.149 | 0.798 | 1.667 | 0.465 |  | |  |  |  |  |
| Liver | 52 | 2.082 | 1.439 | 3.015 | <0.001* | 1.982 | | 1.276 | 3.080 | 0.002* |  |
| Peritoneum | 22 | 1.241 | 0.768 | 2.004 | 0.378 |  | |  |  |  |  |
| Adrenal gland | 7 | 0.705 | 0.309 | 1.604 | 0.404 |  | |  |  |  |  |
| Skin/soft tissue | 7 | 1.876 | 0.816 | 4.309 | 0.138 |  | |  |  |  |  |
| Brain | 10 | 1.098 | 0.556 | 2.167 | 0.788 |  | |  |  |  |  |
| No. of metastatic organs ≥ 2 | 70 | 1.517 | 1.064 | 2.164 | 0.021* | 1.083 | | 0.7111 | 1.648 | 0.711 |  |
| ECOG PS≥3 (vs PS=2) | 55 | 1.312 | 0.907 | 1.898 | 0.149 |  | |  |  |  |  |

Abbreviations: CI, confidence interval; ECOG, Eastern Cooperative Oncology Group; NLR, neutrophil-lymphocyte ratio; OR, odds ratio; PS, performance status; UC, urothelial cancer. *P < 0.05.
